# The London Lancet

**Published:** 1860-05

**Authors:** 


THE LONDON LANCET: A Journal of British and Foreign Medicine, Physiology, Surgery, Chemistry, Literature, Criticism and News.

We have received through the enterprising American Publisher, the above well-established, progressive and popular medical monthly in exchange for the *Examiner*, and we are fully convinced that the "*Lancet* may be said to have special claims upon the profession in this country." The *Lancet* is made up of Lectures by the most eminent physicians and surgeons of the day. 2. Original Essays, Papers and Clinical Contributions. 3. The Mirror of London and Provincial Hospital Practice, and numerous Clinical Records. 4. Leading Articles and Annotations on all scientific and other topics affecting the internal and public relations of the medical profession. 5. Reports of the proceedings of the Medical and other Societies. 6. Home and Foreign Medical Intelligence. 7. Reviews of New Books and Inventions. 8. Miscellaneous Correspondence. 9. Medical Facts, News Items, etc., etc.

All subscriptions addressed to Jas. Herald, Proprietor of the *Lancet*, 12 Ann Street, N. Y.

